# Sex differences in DNA methylation of the cord blood are related to sex-bias psychiatric diseases

**DOI:** 10.1038/srep44547

**Published:** 2017-03-17

**Authors:** Mariana Maschietto, Laura Caroline Bastos, Ana Carolina Tahira, Elen Pereira Bastos, Veronica Luiza Vale Euclydes, Alexandra Brentani, Günther Fink, Angelica de Baumont, Aloísio Felipe-Silva, Rossana Pulcineli Vieira Francisco, Gisele Gouveia, Sandra Josefina Ferraz Ellero Grisi, Ana Maria Ulhoa Escobar, Carlos Alberto Moreira-Filho, Guilherme Vanoni Polanczyk, Euripedes Constantino Miguel, Helena Brentani

**Affiliations:** 1 Brazilian Biosciences National Laboratory (LNBio), Brazilian Center for Research in Energy and Materials (CNPEM), Campinas, Brazil; 2 Institute of Psychiatry, University of São Paulo Medical School, SP, Brazil; 3 Department of Pediatrics, University of São Paulo Medical School, SP, Brazil; 4 Department of Global Health and Population, Harvard School of Public Health, USA; 5 Department of Pathology, University Hospital of Sao Paulo, SP, Brazil; 6 Department of Obstetrics and Gynecology, University of São Paulo Medical School, SP, Brazil

## Abstract

Sex differences in the prevalence of psychiatric disorders are well documented, with exposure to stress during gestation differentially impacting females and males. We explored sex-specific DNA methylation in the cord blood of 39 females and 32 males born at term and with appropriate weight at birth regarding their potential connection to psychiatric outcomes. Mothers were interviewed to gather information about environmental factors (gestational exposure) that could interfere with the methylation profiles in the newborns. Bisulphite converted DNA was hybridized to Illumina HumanMethylation450 BeadChips. Excluding XYS probes, there were 2,332 differentially methylated CpG sites (DMSs) between sexes, which were enriched within brain modules of co-methylated CpGs during brain development and also differentially methylated in the brains of boys and girls. Genes associated with the DMSs were enriched for neurodevelopmental disorders, particularly for CpG sites found differentially methylated in brain tissue between patients with schizophrenia and controls. Moreover, the DMS had an overlap of 890 (38%) CpG sites with a cohort submitted to toxic exposition during gestation. This study supports the evidences that sex differences in DNA methylation of autosomes act as a primary driver of sex differences that are found in psychiatric outcomes.

Epigenetics is a dynamic biological mechanism involved in the modulation of an individual’s genome to environmental stimuli. Much of the epigenome is established during embryogenesis and early gestation and is transmitted in a relatively stable fashion^1^. Among the epigenetic factors, DNA methylation is thought to contribute to stable long-term regulation of gene expression. A large study in families with twins reported methylation differences dependent on genetic factors and environment^2^. Due to the stability, DNA methylation might be related to the development of psychiatric diseases later in life^3^. Several studies pointed out to a correspondence at methylation levels between blood and brain within individuals, suggesting that, although with some restrictions, blood can be used as a source to study psychiatric diseases^4^,^5^.

During perinatal sensitive periods, the environment exerts a critical impact on the maturation of brain structure and function^6^,^7^,^8^,^9^,^10^. It has been well characterized in humans that exposure to external damaging agents, such as smoking, mercury and arsenic during gestation induced epigenetic alterations in newborns^11^,^12^,^13^. Likewise, early life adversity, including pre- and post-natal stress, increased the risk of psycopathologies in animal models^14^. In humans, the Dutch Hunger Winter studies demonstrated the relationship between famine exposure at different times of prenatal development and phenotypic outcomes. Famine exposure around the period of conception was associated with increased risk of adult schizophrenia^15^, and with persistent changes in DNA methylation for *INSIGF, GNASAS, LEP, MEG3, IL10* and *ABCA1*^16^. Furthermore, epidemiological studies indicated that psychological stress such as bereavement, unwanted pregnancies, military invasion and natural disasters increased the risk for the offspring to develop neurodevelopmental diseases later in life, such as schizophrenia, depression and anxiety^17^,^18^,^19^,^20^.

The relation between psychiatric diseases and adverse experience in early life were included in the Developmental Origins of Health and Disease (DOHaD) hypothesis. Most DOHaD diseases have a sex-biased susceptibility^21^,^22^, suggesting that the response to stress is different in males and females. Accordingly, when exposed to stress, males suffer from behavioral problems earlier in development - at one year of age^23^, whilst females show the effects at later life periods^24^,^25^. In addition, there is a clear temporal specificity and sexually dichotomous influence of the effects of prenatal stress^26^,^27^,^28^. Males seem to be more vulnerable to develop schizophrenic, autism and attention-deficit/hyperactivity disorder (ADHD) symptoms in response to perinatal stress, all these were associated with intra uterus growth restriction (IUGR)^29^,^30^.

Animal models also contributed to association between methylation alterations and different response to stress exposition in females and males. For instance, intrauterine exposure to Bisphenol A (BPA), an estrogenic endocrine disruptor, seems to induce DNA methylation changes in male cortex and in female hypothalamus as result of expression changes in *ESR1* and *DNMT1*^31^. In mice, treatment with valproic acid during gestation induced sex-specific morphological alterations at a discreet region of the midbrain, including a higher cell counts in males compared to females^14^. In mice, exposure to stress in the prenatal period caused elevation of *DNMT1* expression levels in females’ placenta, but not males. Contrariwise, males showed response to maladaptive behavioral stress together with change in methylation and expression of the glucocorticoid receptor and corticotropin-releasing factor in the fetal brain^28^.

A correlation between changes in methylation and gene expression were suggested to be involved with the differential brain development^32^. Thus, differences in the regulation of gene expression between females and males could explain the variances in specific brain structure^8^,^27^,^28^,^32^,^33^, cognitive abilities and stress-coping strategies^28^ Some studies already evaluated sex differences at methylation levels between females and males in the cord blood^34^, prefrontal cortex^32^,^35^ and pancreatic isolates^35^, nevertheless, none considered exposition to stress. Whilst it is known that the developing brain has enhanced sex-biased sensitivity to environmental stimuli at specific periods of gestation that contributes to vulnerability to psychiatric diseases, the possibility that DNA methylation differences between females and males could be involved with psychiatric diseases, independently of exposition to stress, remains to be addressed.

To unveil the contribution of stress exposition and the sexual antagonism effects in DNA methylation as well as their association with psychiatric vulnerabilities, we conducted a genome-wide methylation analysis of cord-blood in a well-characterized cohort of children born at term and of adequate weight/size at birth. Adjustments for cell heterogeneity and stress exposure co-variables were performed followed by the identification of differentially methylated CpG sites (DMSs) between females and males. These methylation differences were explored in other cohorts, including brain development, neurodevelopmental disorders and a cohort where children were exposed to stress during gestation.

## Results

### General characteristics of the study population

We used a natural cohort of newborns collected at the University Hospital of São Paulo Medical School, where, typically, only mothers with a not at risk pregnancy do have labor. We randomly collected cord blood samples from 96 individuals. Then, we excluded six pre-term and two post-term newborns, as well as 11 small and seven large for gestational age newborns, since those presentations have already been associated with psychiatric outcomes and stress exposition^36^. The final cohort included 71 cases, comprised of 39 females and 32 males, all with adequate weigh/size for gestational age and born at term. Moreover, to maximize differences in methylation probably related to sexes differences, we corrected for other exposure variables that could interfere with the analysis (Table 1, Supplemental Table 1) explored by this study (females versus males). Although smoking were different between females and males (p = 0.034), only psychiatric disease in father’s family presented a significant value as given by the sva package analysis (Supplemental Figure 1) and was corrected accordingly. In addition, data were adjusted for batch effects and cell type composition. Only as a proof of concept, the sex of each sample was confirmed by methylation levels of sexual chromosomes. The study design was included as a chart showing the analyses flow (Supplemental Figure 2).

### Methylation differences between sexes correlate with gene expression

**Figure 1:**
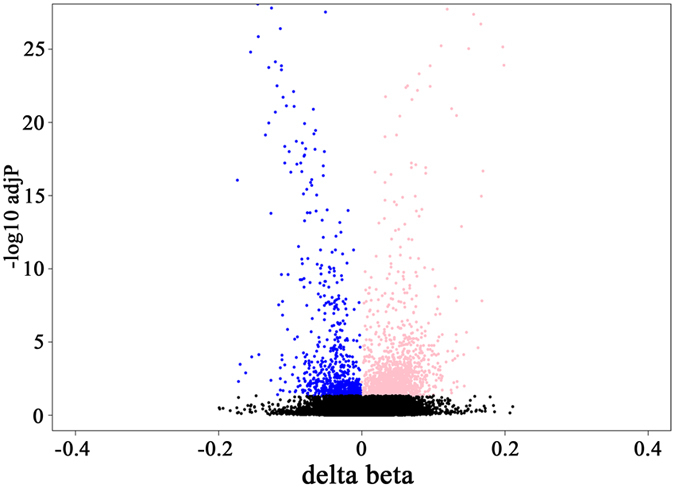
Characterization of the differentially methylated CpG sites between females and males. Volcano plot of the differential methylation analysis with X and Y axes displaying, respectively, the delta-beta values and the log10 of adjusted p-values for each CpG site. CpGs more methylated in females and males are represented in pink and blue, respectively.

To characterize methylation changes in the cord blood of females and males, methylation levels were derived for 464,964 CpG sites, excluding XYS probes. Supervised comparison between sexes identified 2,332 differentially methylated CpG sites (DMSs) with females presenting twice the number of hypermethylated sites compared to males (adjP < 0.05, 1,546 and 786 CpG sites more methylated in females and males, respectively), with 1,499 CpG sites associated to 1,113 genes (Fig. 1; Supplemental Table S2). Upon clustering of the 2,332 CpG sites (Euclidian distance with average linkage), two clusters formed, completely separating both sexes (Supplemental Figure 3).

To evaluate if DMSs were associated with differentially expressed genes between females and males, we used an expression dataset of 2,500 samples from 13 tissues^37^. Excluding genes located in the sexual chromosomes, the 1,113 genes were enrichment among differentially expressed genes between females and males in five tissues, four being brain regions (anterior cingulate cortex, frontal cortex, cerebellum and hippocampus), and the fifth being heart (Supplemental Table 3).

Considering that the 1,113 genes have a sex-biased expression, we used MiSig/WebGestalt to verify if there was an enrichment of binding sites for estrogen and androgen receptors and binding sites located at 2 Kb up or downstream from transcriptional start site (TSS) of each of gene. This analysis resulted in 105 genes regulated by estrogen receptor alpha or beta (five motifs, adjP < 0.05), 24 genes regulated by androgen receptor (three motifs, adjP < 0.05), and ten genes regulated by both transcriptional factors. Then, to explore more distant motifs, we used Opossum^38^ that searched for transcriptional factors target sites located at 5 Kb or 10 Kb up or downstream from TSS of 838 (out of 1,113) genes, revealing no enrichment for *ESR1* or *AR* binding sites (Supplemental Table 4).

### Differentially methylated CpG sites are not randomly distributed within the genome

Females have twice the number of methylated CpGs sites than males, with a non-random distribution in the genome. CpG sites more frequently methylated in females were more often located near transcriptional start sites (TSS1500) and within CpG islands, S_shores and S_shelves. Conversely, CpG sites more frequently methylated in males were enriched within gene bodies and 3′UTR, and were associated with open sea areas (Supplemental Table 5, chi-square test, p < 0.05). Based on the predefined regions, the differentially methylated regions (DMRs) between females and males were distributed as follow: 171 CpG islands (98 and 73 hyper- and hypomethylated in females), 131 promoters (80 and 51 hyper- and hypomethylated in females), 127 genes (64 and 63 hyper- and hypomethylated in females) and 333 genome-wide tiling regions (169 and 164 hyper- and hypomethylated in females) (Supplemental Figure 4, Supplemental Table 6, adjP < 0.05). The top DMRs (adjP < 0.05 and at least 10 CpG sites) are shown in Table 2.

### Differentiated methylated sites between males and females are related to genes associated with normal brain development and neurodevelopment disorders

To gather a global view of the role of these genes, the 1,113 genes were annotated into gene ontology, revealing over-representation of genes related to system development, neurogenesis and neuron differentiation, striated muscle cell differentiation, and single organism behavioral and synaptic transmission. In addition, we found enrichment of genes belonging to the metabolic pathway (60 genes), the MAPK signaling pathway (23 genes), and focal adhesion (19 genes), among others (adjP < 0.01), including the insulin signaling pathway (12 genes). Finally, we observed an enrichment of genes related to neuropsychiatric disorders, stress, cancer, congenital disorders, musculoskeletal including cardiovascular diseases, endocrinological and female and male genitourinary diseases (Supplemental Table 7).

To test if the 1,113 genes were associated with brain development, we searched in the Allen Brain Atlas and found that 930 genes were expressed in at least one of the 26 brain regions (8^th^ post-conception week to 40 years of age). Next, the comparison between the 2,332 DMS and the 7% of CpGs that changes the methylation during brain development^39^ showed an enrichment, represented by 238 CpGs (p < 0.00001, Supplemental Figure 5). Further, we compared to the 521 CpG sites that, in addition to being modulated during brain development, were also differentially methylated between brains of females and males, resulting in 195 common CpG sites (p < 0.00001, Supplemental Figure 5). Finally, extracting data from the only study in schizophrenia that had used the 450 K Beadchip arrays with publicly available data^40^ we searched for enrichment between 2,332 DMS between females and males and the 4,641 differentially methylated CpG sites in brain tissue from schizophrenic cases and controls. The analysis reported an overlap of 116 CpG sites between both studies, indicating an enrichment for CpG sites related to schizophrenia among CpG sites that differ between females and males in cord blood (p = 0.0139, Supplemental Figure 5).

### Differentiated methylated sites between females and males are enriched for psychiatric disorders in a stress exposition model

To further explore the enrichment of DMSs between sexes after correction for stress exposition among genes associated with psychiatric disorders, we compared our DMSs to the differentially methylated CpG sites in the cord blood between females and males exposed to stress (toxic agents) during gestation from an independent cohort. A study from CHAMACOS (a longitudinal birth cohort study of the effects of exposure to pesticides and environmental chemicals on the health and development of Mexican-American children living in the agricultural region of Salinas Valley, CA) assessed the methylation changes related to sex differences in the cord blood of females and males, taking into account batch effects and cell composition. Excluding XYS CpG sites, they found 3,031 DMS (25.7% of the total) located in the autosomes, with most of them (82%) more methylated in females^34^. We compared these to our 2,332 DMS and found 890 (38%) common DMSs, all presenting the same pattern regarding hypo- and hypermethylation.

To assess whether there was an enrichment of genes associated with psychiatric diseases in both data sets, CHAMACOS‘ and ours, we used only those DMSs related to genes. Considering that one gene may have more than one CpG site, and different CpG sites may have opposing methylation differences (hypo- or hypermethylated), depending on their location, we excluded genes represented by more than one CpG site. This analysis resulted in 472 common genes between CHAMACOS‘ and ours, 1,309 and 500 genes belonging exclusively to CHAMACOS‘ or our datasets, respectively. Next, an enrichment analysis for diseases were performed with full datasets followed by the enrichment analysis with common and exclusive genes of the two cohorts that showed that, considering all diseases, most of the psychiatric diseases were enriched in all datasets, although there were differences in the ranking as exemplified by the top ten diseases (Supplemental Table 8, diseases and schizophrenia).

**Figure 2:**
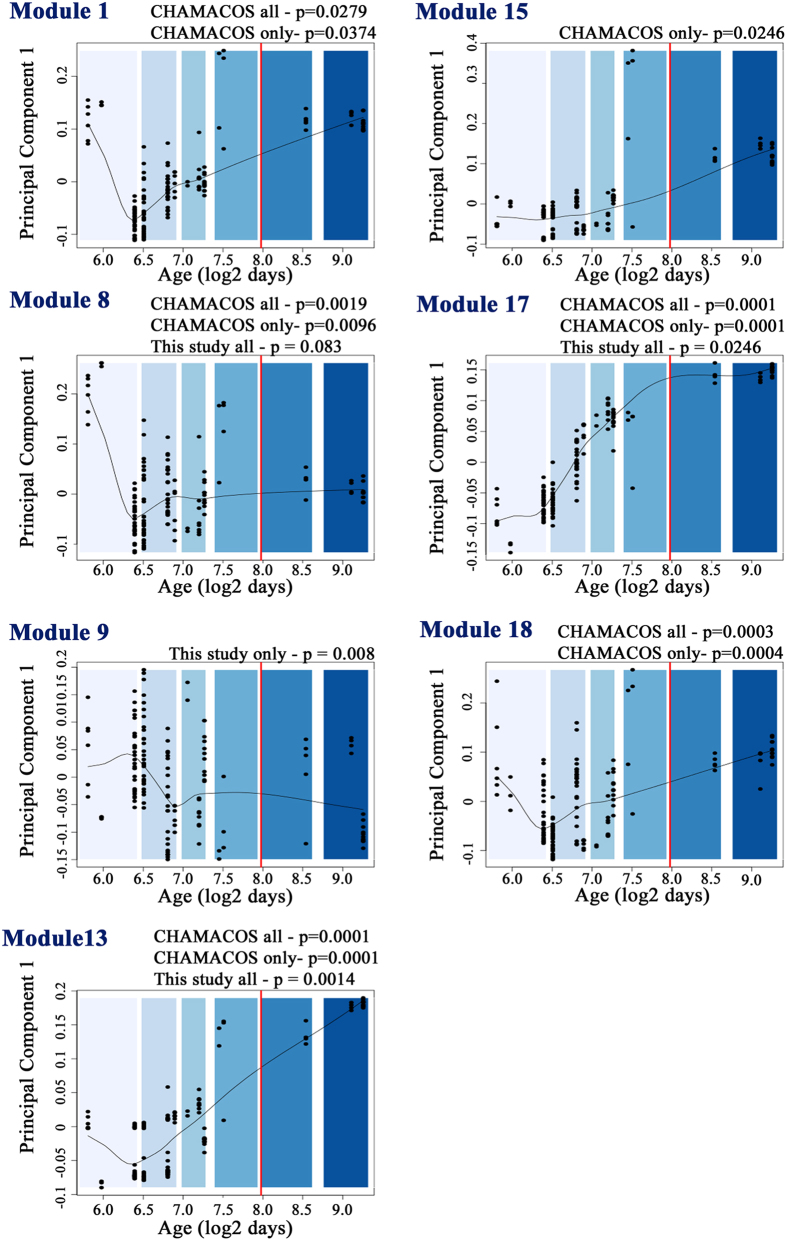
Enrichment analysis for the modules from Parikshak *et al*.^41^, that identified genes co-expressed during cortical development. Graphs show the distribution of genes related to the studies in enriched modules (p < 0.05). Scatter smooth of module eigengene values was defined by Parikshak *et al*.^41^ at different stages of neocortical development. Different periods of lifetime are scaled from light to dark blue colors: early foetal, early-mid foetal, late midfoetal, late foetal, neonatal/early infancy and late infancy. The red line indicates time of birth. Sets of genes enriched for the modules are displayed below of each graph.

These gene sets were tested for enrichment in modules of co-expressed genes along human neocortical development^41^ (Supplemental Table 9). M8, M13 and M17 were enriched within the full datasets and among common genes. M8 is comprised of genes more expressed in the beginning of gestation; genes from M13 present a decreasing expression until postconception week (PCW) 16, from which point present an increasing pattern; and genes from M17 increase after PCW 16 until birth, from which point it seems to reach a plateau. Genes exclusively from this study (n = 500) were enriched for M9 (p = 0.0009), which contains genes highly expressed in the beginning of gestation that decrease expression levels at around 12^th^ week whereas genes exclusively from CHAMACOS‘ (n = 1,309) were enriched for M15 (p = 0.0246), that presents an increasing level of expression during the whole neocortical development. Genes found only by CHAMACOS study (total or exclusive genes) were enriched for M1, that present a decreasing expression until week 12^th^, from which point present a constant increasing expression levels (Fig. 2).

## Discussion

Differences underlying sexes have been associated with distinct incidence and severity of a group of psychiatry diseases, although the mechanisms are not well understood, moreover, differential methylation has been involved with this sex bias prevalence of psychiatry disorders. The immune system response during certain periods of development has sex-specific effects on regulation of various neurotransmitters important for the stress response throughout life, with consequences for physical and psychiatric disorders in later life^36^. Besides, there is a clear sex dimorphism in structures during brain development that were implicated to a sex-bias vulnerability to psychiatric disorders independent of stress exposition^42^. The potential underlying mechanisms driving these sex differences regarding stress responses and their relevance to disease were deeply discussed. The authors concluded that the continued focus and appreciation for how sex differences in stress responses may predict disease risk and resiliency is critical for developing preventive strategies and treatments^43^. In this study, we conducted a genome-wide DNA methylation profile of cord blood of a well-characterized cohort composed of babies born at term and with adequate weight, after correcting for stress exposition factors that could interfere with DNA methylation. We showed that differential methylation between females and males found in the cord blood can represent gene expression differences in tissues with sex dimorphism.

The DMS were equally distributed across all 22 autosomes, as previously described^34^, but their locations were not random. Whilst females had more methylated CpGs associated with repressed gene expression, males had more methylated CpGs in the open sea, which is more often positively correlated with expression^44^,^45^.

Pathways enriched for the 1,113 genes related to the DMS between sexes included metabolic and MAPK pathways. Changes in the metabolism of 1-carbon and methyl donor availability in the fetal liver, a major metabolic organ, were suggested to have a sex influence in a subset of genes^46^. Several pathways related to muscle were over-represented, such as the hypertrophic cardiomyopathy and vascular smooth muscle contraction pathways. The development and establishment of musculature is thought to be a major difference between adult males and females. At molecular level, these differences seem to be present already at birth. Accordingly, cardiovascular and musculoskeletal disorders were enriched in the diseases analyses.

The enrichment analysis revealed a possible role of our gene set to the brain as CpG sites and genes are modulated during brain development on top of being differently methylated between sexes^39^. Furthermore, these genes have been associated to psychiatry diseases as demonstrated by the enrichment of the 2,332 DMSs between females and males among the 4,641 differentially methylated CpG sites in brain tissue between patients with schizophrenia and controls^40^. Among the common genes, *SHANK* genes participate in synaptic formation, including in *SHANK2* and *SHANK3*, in which mutations were described in a wide spectrum of neurodevelopmental and neuropsychiatric diseases^47^.

When analyzing the near and distant motifs for sex hormones, we suggested that: (1) the differential methylation found in CpG sites could interfere with the binding in motifs close to TSS of transcriptional factors classically related to females and males and; (2) other factors that are not exclusively associated with the regulation performed by sexual hormones are contributing to differentially expressed and methylated genes between females and males. Also, most of the DMS (66%) showed higher DNA methylation in females compared to males. It has been reported that females have higher methylation levels than males, although most studies used the X and Y chromosomes in their analyses, where a substantial portion of the DMSs are located^34^. Importantly some of these studies were performed in tissues other than cord blood, such as brain or pancreas, and with older subjects, that present changes in methylation because of the aging process^35^,^48^,^49^.

X dosage and the presence of Y chromosome exerts a role for the methylation patterns on the autosomes. One X was associated to hypomethylation on the autosomes but the presence of Y changes this pattern, suggesting the existence of an interaction between X and Y dosages^50^, meaning that, in addition to the organization effects of gonadal hormones, the sex chromosomes contribute to the differential methylation between females and males and these differences might contribute the sex bias psychiatric vulnerabilities, agreeing with a previous published discussion^43^.

The importance of considering the sex differences relies on the different response from females and males to stress to maintain homeostasis, which may have different consequences for the long-term responses to perturbations from the environment^33^. Prenatal stress can induce long-term neurodevelopmental diseases, particular those related to the hypothalamic-pituitary-adrenal (HPA) response to stress^51^. This exposure to maternal stress may alter the stress-vulnerability of the embryo leading to an increased risk of psychopathology^36^. The cohort from CHAMACOS was subjected to toxic stress (e.g. exposure to pesticides)^34^ whilst our study excluded factors already associated with psychiatric vulnerabilities (not born at term and not adequate size/weight for gestational age); and corrected for stress factors covariables that potentially interfere with DNA methylation. Psychiatric disorders were associated to genes from both studies. Nevertheless, comparing both data, we found 890 overlapping CpG sites, with our methylation differences (delta-beta) being smaller than theirs were, suggesting that exposition to stress might increase these differences. Comparing both datasets to the co-expressed gene modules during neocortical development revealed that some modules were similarly enriched. In addition, genes from these modules (M8: *SOX1, CDKN2C, TRIP10, SALL1, EFNA4, PARD3B, CDH23, PRDM16, NEK9, C1orf61, HSPB1, CCDC8, FLNB* and *TEAD3*; M13: *RTKN, SYN2, SYNGR1, NRXN3, PRSS16, CPLX1, ZNF365* and *SLC17A7*; *M17: CYP1A1, GRIN1, GRIN2D, PRPH, CSMD1, BTBD9, UPP2*, and *USP12*) were found among genes enriched for the overrepresented psychiatric diseases. For instance, *SYN2*, more methylated in females (cg10245988, island), encodes a neuron-specific phosphoprotein that selectively binds to small synaptic vesicles in the presynaptic nerve terminal. Polymorphisms in this gene were associated with abnormal presynaptic function and increased risk for related neuronal disorders, including autism, bipolar disorder and schizophrenia^52^,^53^,^54^. SNPs in *UPP2* were associated with a sex-bias factor in children diagnosed with autism spectrum disorder^55^. Nonetheless, the enrichment analysis of specific datasets reported different groups of genes regarding their expression levels during neurodevelopment, suggesting specific contributions.

We are aware that this study has limitations. First, we controlled for a limited number of stress factors and cannot guarantee that the methylation analyses were not influenced by unexplored stress exposition factors. Second, we could not measure contamination of maternal cells and thus, did not correct for it. Third, comparisons between data from cord blood and brain were not performed with samples collect from the same individuals. Fourth, we used only exposure to pesticides and environmental chemicals as a model toxic stress. Finally, we found only one study with publicly available data that used the H450K to evaluate DNA methylation in the brain of patients with schizophrenia representing psychiatric diseases found enriched in our analyses.

This study corroborated previous findings from the literature that showed that methylation differences between females and males in the cord blood methylation are associated with brain developing tissues. We describe there that genes related to this differential methylation are enriched for genes previously associated with psychiatric diseases, even after correcting the methylation analysis for stress exposition covariables, in additional to the technical effects. These differences are not solely explained by gonadal hormone effects, but also by sex chromosomes effects on the autosomes. Moreover, they present specific pattern of gene co-expression organized in modules of neocortical development, which implies temporal specific effects.

## Material and Methods

### Sample collection

Samples came from the Western region of Sao Paulo city, where 6,200 women were enrolled between 26^th^ and 34^th^ week gestation between 2012–2014 (Regiao Oeste Cohort, ROC). For this study, a questionnaire was administered to mothers to evaluate levels of stress and toxic exposures during gestation (smoke, use of drugs). These children are being followed up to gather additional information related to neurodevelopmental diseases and behavioral phenotypes as a continuum of this study; this will not be explored in here due to the relatively small time-lapsed. We collected blood from the umbilical cord of 96 women that delivered at the University Hospital of Sao Paulo. All women gave written informed consent to participate in the study. The study itself, as well as the use of samples, were conducted with ethical approval granted by the Hospital’s Internal review board – CEPHU under protocol number 1076/10, with all experiments performed in compliance with the Helsinki Declaration.

### Sample preparation and quality control

DNA was isolated from umbilical cord blood samples using QIAamp DNA Blood Midi Kit (Qiagen). NanoDrop (Thermo Fisher Scientific) and Qubit (Thermo Fisher Scientific) was used to assess DNA purity and quantity. DNA quality was checked by electrophoresis in 0.8% agarose gels followed by bisulfite conversion whereby 1 ug DNA was treated using the EZ DNA Mehylation kit (Zymo Research Corp), following manufacture recommendations.

High quality bisulfite-converted DNA from 96 samples were hybridized in the Human Methylation 450 BeadChip microarrays (HM450K, Illumina), following the Illumina Infinium HD methylation protocol. Both experiments were processed by Deoxi Biotecnologia (www.deoxi.com) according to manufacturer’s instructions. Raw data were extracted by the iScan SQ scanner (Illumina) using GenomeStudio software (v.2011.1), with the methylation module v.1.9.0 (Illumina), into IDAT files, which were used for further analyses. The HM450K platform interrogates DNA methylation levels of 485,577 *loci* distributed across the genome at single-nucleotide resolution. Probes were annotated according regarding their nearest genes using FDb.InfiniumMethylation.hg19 package with an additional annotation for those probes that were located at more than one gene, Hg19 coordinates from UCSC were selected. This resulted in 20,461 unique entrezID genes. The methylation levels for each CpG probe were provided in beta-values (0 indicating unmethylated CpGs, and 1, fully methylated CpGs). All analyses were performed in R environment using Bioconductor packages (http://www.bioconductor.org). All samples had high quality data passing QC using standard parameters.

### Genome-wide methylation analysis

The RnBeads package^56^ was applied to the dataset. Unreliable measurements were identified using Greedycut (p > 0.05) that removed 1,478 probes. Probes located in SNPs (n = 4,776) as well those located in specific contexts (n = 5,926 – non-CpG sites as well as additional SNPs) were also excluded. Samples were retained only if >99% of sites assayed had detection p > 0.05. The background was corrected using noob method, which is based on a normal-exponential convolution using out-of-band probes^57^. Signal intensities values from probes type I and II were normalized using SWAN method that adjusts the intensities based on a quantile approach^58^. Probes located in the sexual chromosomes were filtered out remaining 464,964 probes for analyses.

To quantify methylation differences resultant from cellular composition of the blood, a purified dataset containing six blood cell types was used as ref. 59 to estimate the blood cell type contribution for each cord blood sample. Based on the estimated percentages of each cell type, a projection of the methylation levels for each cord blood sample was used in the limma based analysis of differential DNA methylation, as implemented by RnBeads^56^. Variables from the questionnaire were tested for association with the main group comparison (female versus male) indicating that only psychiatric disease in the father family was acting as co-variables. Moreover, adjustment for technical effects (e.g. batch effects) and cell type composition were performed before the differential methylation analyses.

To identify differentially methylated CpG sites (DMSs), the M-values (loggit of B-values) were used employing an empirical Bayesian framework linear model from limma^60^. CpG sites with FDR adjusted p-value (adjP) <0.05 were selected as differentially methylated. RnBeads also identified differentially methylated regions (DMRs) in pre-defined CpG islands, promoters, genes and genome-wide tiling regions, that exhibit DNA methylation changes between females and males (FDR adjP < 0.05).

### *In silico* functionally exploration of data

Functional and disease enrichment analysis were performed in WebGestalt^61^ using the whole genome as background. Significant values were considered to be those features comprised by at least 10 genes and adjusted p-value (adjP) <0.01 adjusted by the multiple test as given by Benjamini–Hochberg procedure.

CpGs were compared to differentially methylated CpG sites found by three studies with publicly available data^34^,^40^,^62^ using all probes from 450 K BeadChip as background. In addition, genes associated to DMSs were compared to 17 modules comprised of co-expressed genes during human cortical development^41^. For both cases, we applied the Modular Single-set Enrichment Test (MSET)^62^, which assess enrichment for a gene or CpG site (here named as feature) list of interest within a set of features using a randomization testing. First, the probability of our list of features being significantly represented in each module were compared to 10,000 simulated sets of features generated randomly from the 450 K Beadchip array (for DMS) or RefSeq (for genes) as background. Then, a p-value was calculated based on the number of simulated randomized sets that contains equal or higher number of features belonging to our list of features in the list generated by each of the three studies. We considered as significant, those comparisons with a p-value < 0.05. For the comparison with modules of genes co-expressed during neocortex development, the module eigengene values from the original study^41^ were plotted in a scatter smooth plot for the significant modules.

### Data access

The HumanMethylation450 BeadChip data set from this study is available in NCBI Gene Expression Omnibus (GEO) under accession number GSE85042.

## Additional Information

**How to cite this article:** Maschietto, M. *et al*. Sex differences in DNA methylation of the cord blood are related to sex-bias psychiatric diseases. *Sci. Rep.* **7**, 44547; doi: 10.1038/srep44547 (2017).

**Publisher's note:** Springer Nature remains neutral with regard to jurisdictional claims in published maps and institutional affiliations.

## Supplementary Material

Supplementary Information

Supplementary Table 1

Supplementary Table 2

Supplementary Table 3

Supplementary Table 4

Supplementary Table 5

Supplementary Table 6

Supplementary Table 7

Supplementary Table 8

Supplementary Table 9

## Figures and Tables

**Table 1:** Characteristics of the cohort and factors evaluated as covariables of the analyses.

	Male (n = 32)			Female (n = 39)			P value
	n	%	Mean (±SD)	n	%	Mean (±SD)	
**Mothers**							
-**Age (years)**			26.9 (5.9)			29.4 (7.0)	0.133
≤18	0			1	2.56		
19–35	29	90,7		31	79.5		
≥36	3	9.3		7	17.9		
-**Educational level**							0.840
Unlettered	2	6.5		1	2.6		
Elementary school	11	35.5		14	35.9		
High school	17	54.8		20	51.3		
College	0			1	2.6		
Unknown	1	3.2		3	7.7		
**Income** (**Minimal wages**)			4.0 (1.0)			4.5 (0.8)	0.399
≤2	9	29.0		18	46.1		
3–4	11	35.5		14	35.9		
≥5	7	22.6		3	7.7		
Unknown	4	12.9		4	10.3		
-**Type of delivery**							0.815
Normal	17	57.7		21	53.9		
Cesarean section	13	43.3		18	46.1		
-**Planned gestation**	26	83.9		34	87.2		0.727
-**Self**-**reported environmental factors**							
-**Stress during gestation**	15	48.4		20	51.3		0.525
-**Feelings during gestation**							0.546
Most time, positive	18	58.1		18	46.2		
Soft stress	4	12.9		10	25.6		
Moderate stress	6	19.4		5	12.8		
Abortion was considered	2	6.5		5	12.8		
Severe stress	1	3.1		1	2.6		
-**Psyquiatric drugs during pregnancy**	0			2	5.1		0.201
-**Smoking during gestation**	2	6.3		10	25.6		0.034
-**Drugs/alcool during pregnancy**	12	38.7		9	23.1		0.156
**Neonates**							
-**Gestational age** (**weeks**)	30		39.5 (1.4)	39		39.4 (1.2)	0.876
-**Birth Weight** (**g**)	30		3359.2 (396)	39		3298.2 (321)	0.482
-**Length**	30		49.3 (1.7)	39		48.1 (1.8)	0.009
-**Cephalic circumference**	30		34.7 (1.3)	38		34.2 (1.0)	0.123
-**Thoracic circumference**	30		33.2 (1.4)	38		33.2 (1.4)	0.963
-**Abdominal circumference**	30		31.9 (1.6)	38		32.3 (1.8)	0.361

**Table 2:** Differentially methylated regions in predefined genomic regions with adjusted p-value < 0.05 and containing at least 10 CpG sites.

ID	Chromosome	Start	End	Gene symbol	entrezID	Mean difference	Combined adjP	CpG sites
**CpG islands**								
20510	chr17	20798958	20800112			−0,046	3,79E-03	11
22902	chr19	6495193	6496225			−0,017	1,94E-03	10
25607	chr21	27106815	27108211			−0,003	2,13E-03	10
4617	chr3	57993839	57994704			0,007	1,11E-02	12
3768	chr2	208576002	208577106			0,016	1,68E-03	10
4494	chr3	49394856	49395942			0,017	1,08E-02	10
18564	chr15	101094521	101099493			0,020	7,65E-03	13
18889	chr16	2569560	2571071			0,024	1,22E-02	13
16491	chr13	25874995	25876200			0,026	3,76E-02	12
7248	chr5	149546028	149546988			0,058	2,20E-03	10
**Promoters**								
ENSG00000237268	chr7	56515569	56517568	NA	NA	−0,047	9,62E-03	13
ENSG00000205212	chr17	20798954	20800953	CCDC144NL	339184	−0,042	1,02E-02	12
ENSG00000155918	chr6	150346169	150348168	RAET1L	154064	−0,020	3,75E-02	11
ENSG00000136068	chr3	57992627	57994626	FLNB	2317	0,007	1,39E-02	12
ENSG00000163249	chr2	208574764	208576763	CCNYL1	151195	0,017	1,91E-03	10
ENSG00000162066	chr16	2568858	2570857	AMDHD2	51005; 752014	0,026	1,21E-02	13
ENSG00000139496	chr13	25874162	25876161	NUPL1	9818	0,027	4,45E-02	13
ENSG00000157911	chr1	2344737	2346736	PEX10	5192	0,040	8,61E-03	13
ENSG00000113722	chr5	149544858	149546857	CDX1	1044	0,046	4,24E-02	10
**Genes**								
ENSG00000189136	chr15	85070012	85114447	UBE2Q2P1	388165	−0,022	9,38E-05	10
ENSG00000120705	chr5	137841784	137878989	ETF1	2107	−0,009	1,24E-02	10
ENSG00000164754	chr8	117858174	117887105	RAD21	5885	−0,008	3,62E-02	12
ENSG00000100902	chr14	35747839	35786699	PSMA6	5687	−0,004	2,32E-02	12
ENSG00000154727	chr21	27106881	27144771	GABPA	2551	−0,003	4,83E-02	15
ENSG00000154723	chr21	27088815	27107984	ATP5J	522	−0,001	3,91E-02	14
ENSG00000176714	chr2	27848506	27851879	CCDC121	79635	0,016	6,56E-05	12
ENSG00000241186	chr3	46616045	46668033	TDGF1	6997	0,016	7,33E-03	18
ENSG00000196781	chr9	84198598	84304220	TLE1	7088	0,030	5,00E-05	16
ENSG00000110243	chr11	116660083	116663136	APOA5	116519	0,034	1,68E-02	10
ENSG00000163749	chr4	77234154	77343021	CCDC158	339965	0,034	3,55E-02	12
ENSG00000175773	chr11	130184888	130263688	NA	NA	0,039	7,27E-05	14
ENSG00000121410	chr19	58856544	58864865	A1BG	1	0,050	1,42E-02	12
ENSG00000268895	chr19	58859117	58866549	A1BG-AS1	503538	0,054	2,79E-03	12
**Genome**-**wide tiling regions**								
258039	chr7	56515001	56520000			−0,046	2,44E-02	15
278217	chr7	157405001	157410000			−0,044	4,02E-02	14
504201	chr17	20795001	20800000			−0,039	2,28E-02	13
478486	chr15	85110001	85115000			−0,020	6,65E-04	11
533197	chr19	6495001	6500000			−0,014	1,94E-02	12
41882	chr1	209405001	209410000			−0,010	1,88E-02	10
447146	chr14	35760001	35765000			−0,005	4,10E-02	10
55422	chr2	27850001	27855000			0,011	2,56E-02	20
91567	chr2	208575001	208580000			0,014	1,66E-02	12
481683	chr15	101095001	101100000			0,021	2,99E-03	11
340633	chr10	22765001	22770000			0,024	1,52E-02	11
96502	chr2	233250001	233255000			0,027	4,67E-02	11
107815	chr3	46615001	46620000			0,032	5,47E-03	10
137680	chr3	195940001	195945000			0,033	2,59E-02	13
507646	chr17	38020001	38025000			0,038	2,43E-03	11
213529	chr6	5085001	5090000			0,039	4,10E-02	14
206237	chr5	149545001	149550000			0,045	2,93E-02	13
549738	chr20	30070001	30075000			0,047	3,07E-02	12
66673	chr2	84105001	84110000			0,050	2,35E-08	11
543670	chr19	58860001	58865000			0,065	7,02E-04	10
