# Supplementary material for: Sex differences in DNA methylation of the cord blood are related to sex-bias psychiatric diseases
Source: Sci Rep. 2017 Mar 17;7:44547. doi: 10.1038/srep44547 (PMC5355991; doi:10.1038/srep44547)
Supplement: Supplementary Information [file srep44547-s1.pdf]

Sex differences in DNA methylation of the cord blood are related to sex-bias psychiatric diseases

Mariana Maschietto[*], Laura Caroline Bastos[†], Ana Carolina Tahira[†], Elen Pereira Bastos[†], Veronica Luiza Vale Euclydes[†], Alexandra Brentani[‡], Günther Fink[þ], Angelica de Baumont[†], Aloísio Felipe-Silva[¶], Rossana Pulcineli Vieira Francisco[#], Gisele Gouveia[†], Sandra Josefina Ferraz Ellero Grisi[‡], Ana Maria Ulhoa Escobar[‡], Carlos Alberto Moreira-Filho[‡], Guilherme Vanoni Polanczyk[†], Euripedes Constantino Miguel[†], Helena Brentani[†]

*Brazilian Biosciences National Laboratory (LNBio), Brazilian Center for Research in Energy and Materials (CNPEM), Campinas, Brazil, [†]Institute of Psychiatry, University of São Paulo Medical School, SP, Brazil, [‡]Department of Pediatrics, University of São Paulo Medical School, SP, Brazil, [þ]Department of Global Health and Population, Harvard School of Public Health, USA, [¶]Department of Pathology, University Hospital of Sao Paulo, SP, Brazil, [#] Department of Obstetrics and Gynecology, University of São Paulo Medical School, SP, Brazil.

Supplemental files

Supplemental Figure 1 - Heatmap displaying the results of permutation tests performed for associations of the covariables, as given by RnBeads package. Significant p-values (p<0.01) are displayed in pink background and non-significant values are represented by blue boxes

Supplemental Figure 2 - Chart showing the work-flow of the analyzes performed in this study.

Supplemental Figure 3 - Non-supervised hierarchical clusterization (Pearson correlation with complete linkage based on the methylation levels of the 2,332 differentially methylated CpG sites between females and males. Pink: females, light blue: males.

Supplemental Figure 4 - Scatter plot of groupwise mean DNA methylation levels across CpG islands, promoters, genes and genome-wide tiling with differentially methylated regions presenting adjP<0.05 highlighted in red. Point density is shown as blue shading. X and Y axis represent mean beta-values of females and males, respectively.

Supplemental Figure 5 - Probability density of simulations. Density curve showing 10.000 randomly generated simulated groups of 2,332 CpGs using all 450K probes as background. Blue line indicate whether the number of matches found by our study fall with respect to this distribution. A line far to the right tail of the curve reflects a very low probability that the number of disease associated genes detected in the true results occurred by chance. Enrichment analysis for **A**. Brain development; **B**. CpG sites modulated during brain development and differentially methylated between brains of females and males. **C**. differentially methylated between CpG sites between brains from schizophrenic patients and controls.

Supplemental table 1 – Detailed characteristics of the cohort used in the study.

Supplemental table 2 – The 2,332 differentially methylated CpG sites (adjP<0.05) between females and males. CpG sites common to CHAMACOS' study are labeled in red.

Supplemental table 3 – Genes with sex-bias expression in 13 tissues (extracted from Mayne et al, 2016).

Supplemental table 4 – Enrichment analysis of binding sites of transcription factors ER and AR using Webgestalt (background: human genome, at least 2 genes)

Supplemental table 5 - Distribution of the 2,332 differentially methylated CpG sites regarding their genomic location across the genome. Blue indicates the significant features, as given by Chi-square distribution test.

Supplemental table 6- Differentially methylated regions for CpG islands, promoters, genes and genome-wide tiling are displayed in different sheets. Using predefined genomic regions, the uncorrected, CpG-specific p-values within a given region were combined using an extension of Fisher's method resulting in single aggregate P value for each region, and the aggregate P values are subjected to multiple-testing correction using the FDR method.

Supplemental table 7 - Functional enrichment analyses: Biological Processes from Gene Ontology, cellular signaling pathways from KEGG and Diseases (shown in different spreadsheets) performed using WebGestalt using the whole genome as background. The enriched pathways as well as relevant biological processes related to the 1.113 genes associated with the differentially methylated CpG sites between females and males are shown.

Supplemental table 8 – Diseases enrichment analyses performed in WebGestalt using the whole genome as background. Diseases: Lists used were comprised of 472 common genes between CHAMACOS' and this datasets, 500 and 1,309 genes belonging exclusively to this or CHAMACOS' datasets, respectively. Psychiatric diseases are labeled in red. Schizophrenia: list of 20 genes related to the DMS, which were enriched for schizophrenia. If genes were found only by this study or also by CHAMACOS study was pointed out in the table.

Supplemental table 9 – Enrichment analysis for the modules from Parikshak et al. (2013), that identified genes co-expressed during cortical development, using genes represented in the Illumina 450K Beadchip arrays as background.

Supplemental Figure 1

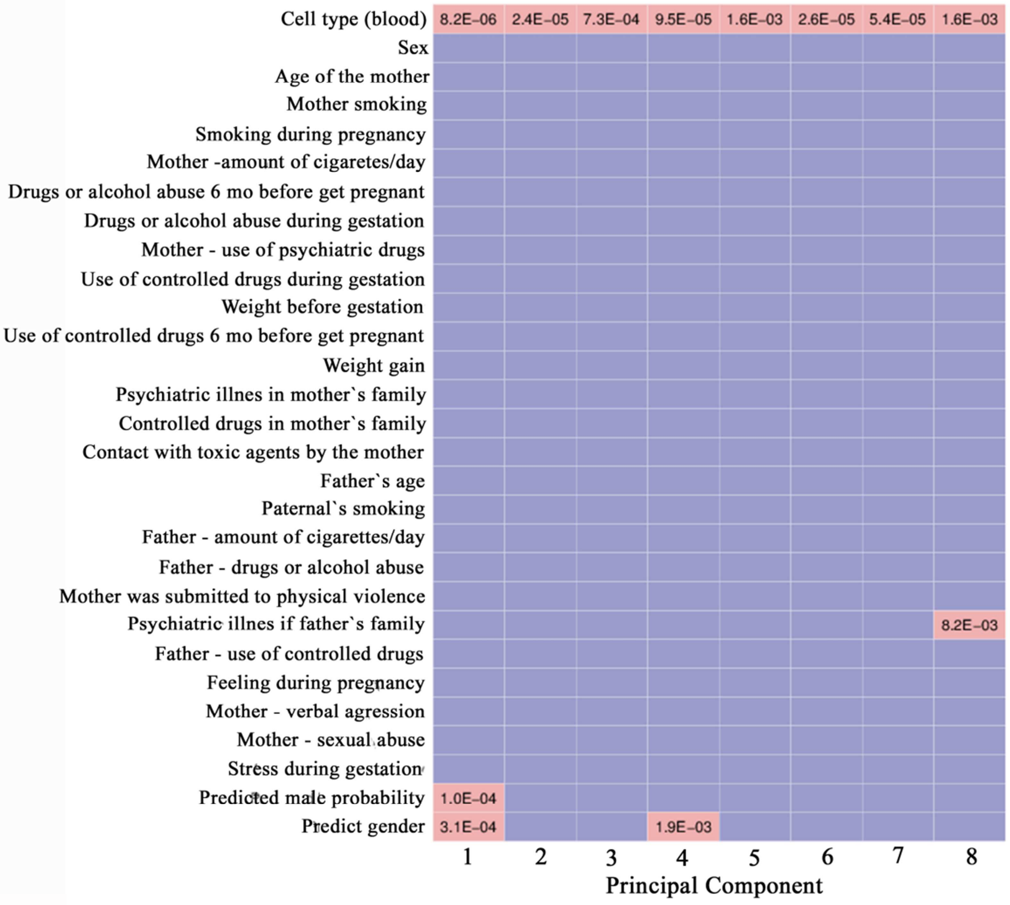

Heatmap displaying the results of permutation tests performed for associations of the covariables, as given by RnBeads package. Significant p-values (p<0.01) are displayed in pink background and non-significant values are represented by blue boxes

Supplemental Figure 2

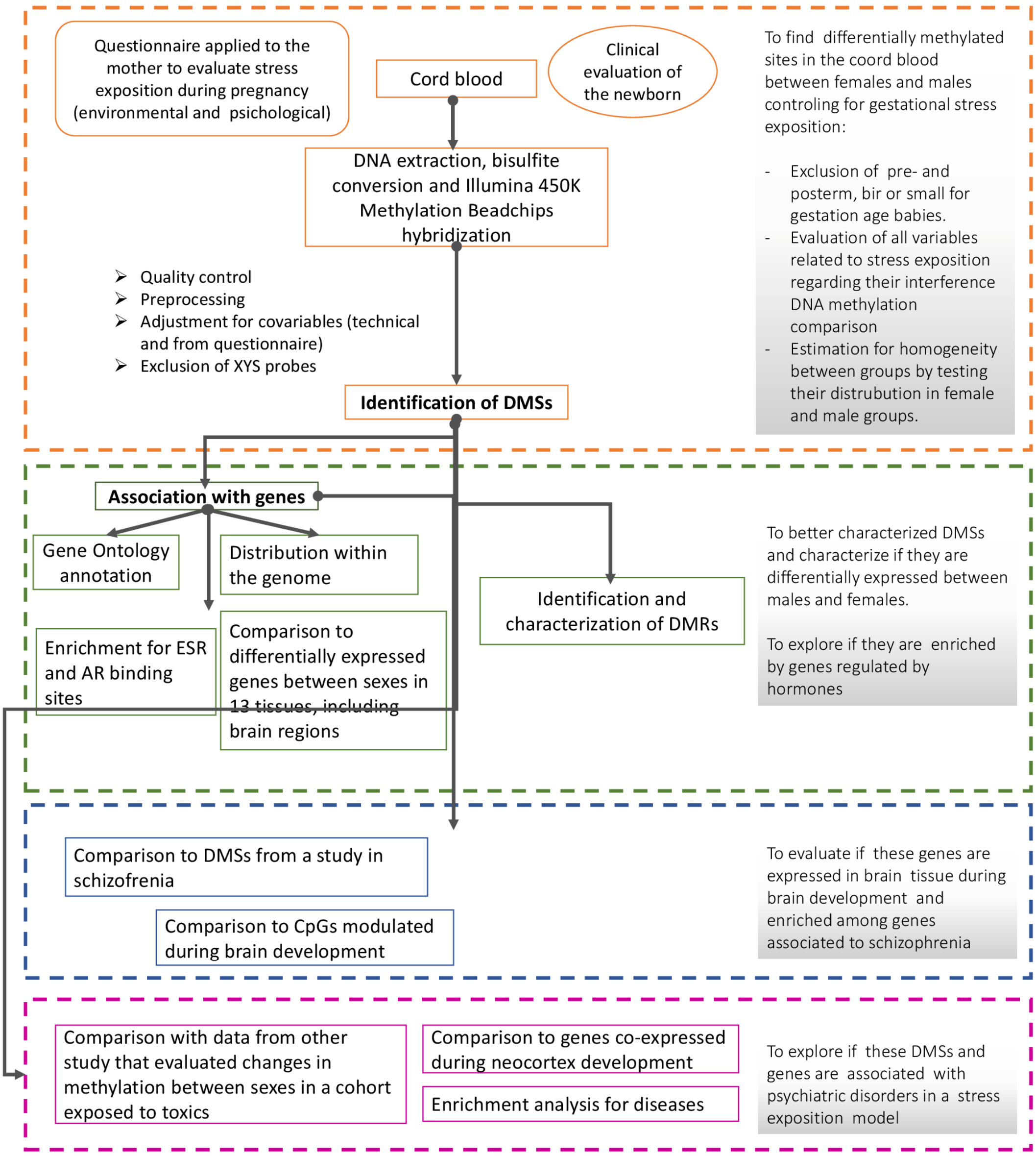

Chart showing the work-flow of the analysis performed in this study.

Supplemental Figure 3

26/01/2017 Supplemental Figure 3

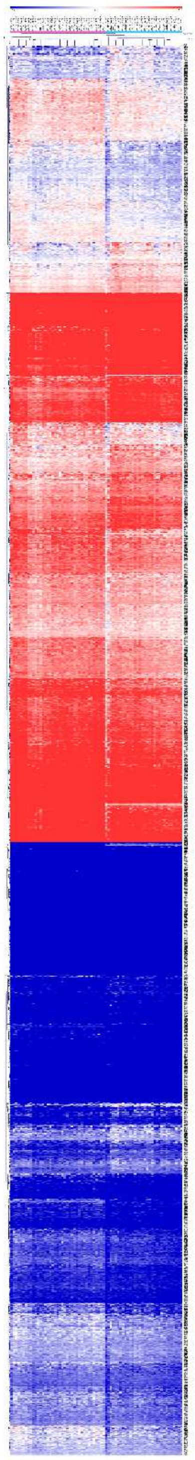

https://drive.google.com/file/d/0B2f4m3B7DFQUSXlvRzBwV09YTFU/view 1/1

Non-supervised hierarchical clusterization (Pearson correlation with complete linkage based on the methylation levels of the 2,332 differentially methylated CpG sites between females and males. Pink: females, light blue: males.

Supplemental Figure 4

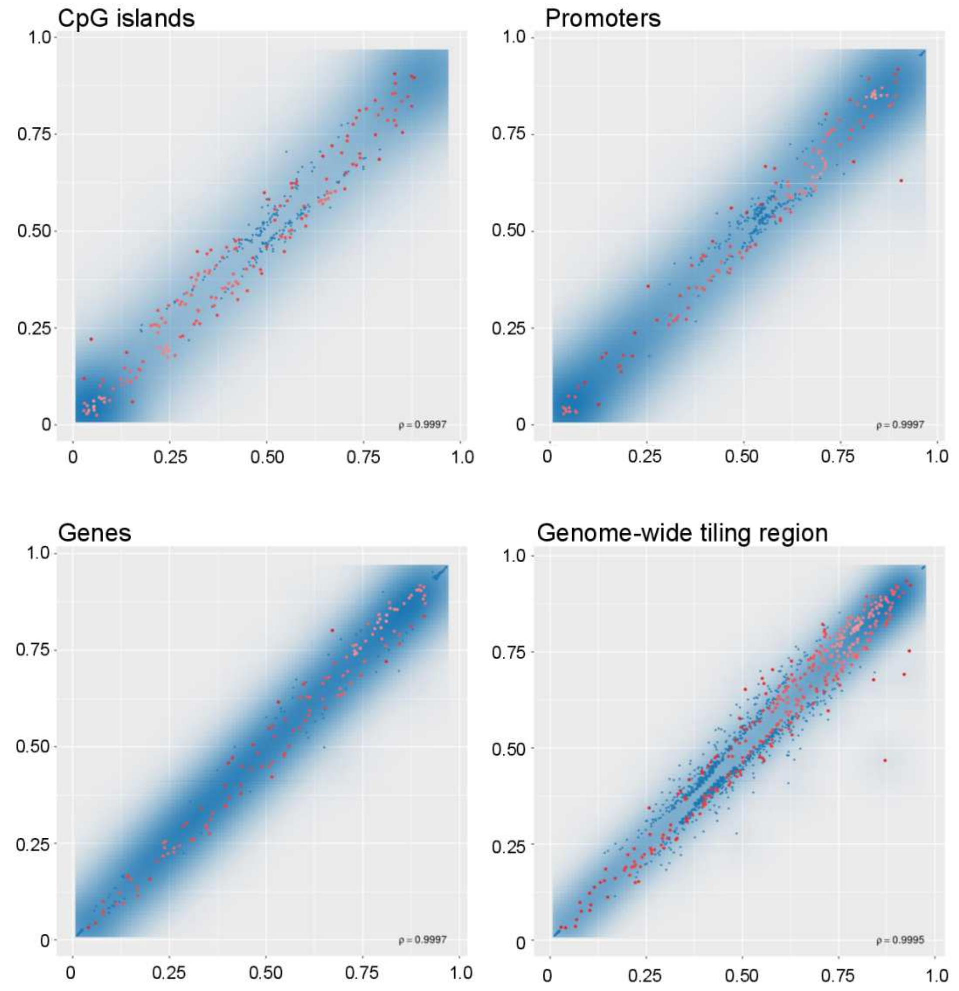

Scatter plot of groupwise mean DNA methylation levels across CpG islands, promoters, genes and genome-wide tiling with differentially methylated regions presenting adjP<0.05 highlighted in red. Point density is shown as blue shading. X and Y axis represent mean beta-values of females and males, respectively.

Supplemental Figure 5

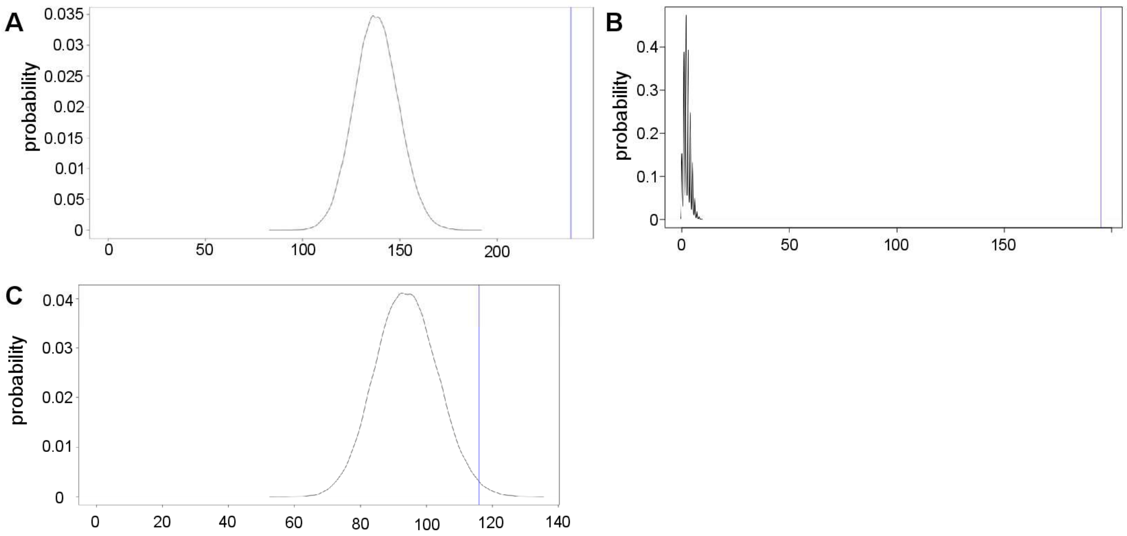

Probability density of simulations. Density curve showing 10.000 randomly generated simulated groups of 2,332 CpGs using all 450K probes as background. Blue line indicate whether the number of matches found by our study fall with respect to this distribution. A line far to the right tail of the curve reflects a very low probability that the number of disease associated genes detected in the true results occurred by chance. Enrichment analysis for **A**. Brain development; **B**. CpG sites modulated during brain development and differentially methylated between brains of females and males. **C**. differentially methylated between CpG sites between brains from schizophrenic patients and controls.
